# Identification of enhanced cytokine generation following sepsis. Dream of magic bullet for mortality prediction and therapeutic evaluation

**Published:** 2010

**Authors:** H. Hamishehkar, M.T. Beigmohammadi, M. Abdollahi, A. Ahmadi, A. Mahmoodpour, M.R. Mirjalili, R. Abrishami, M.R. Khoshayand, K. Eslami, M. Kanani, M. Baeeri, M. Mojtahedzadeh

**Affiliations:** 1 Department of Clinical Pharmacy, Faculty of Pharmacy and Pharmaceutical Sciences Research Center, Tehran University of Medical Sciences, Tehran; 2 Department of Clinical Pharmacy, Faculty of Pharmacy, Tabriz University of Medical Sciences, Tabriz; 3 Department of Anesthesiology and Critical Care Medicine, Imam Khomeini hospital; 4 Faculty of Pharmacy and Pharmaceutical Sciences Research Center; 5 Department of Anesthesiology and Critical Care Medicine, Sina hospital; 6 Department of Food and Drug Control, Faculty of Pharmacy, and Pharmaceutical Sciences. Research Center, Tehran University of Medical Sciences; 7 Deputy of Food and Drug, Ministry of Health and Medical Education, Tehran, Iran

**Keywords:** TNF-α, IL-1β, IL-6, APACHE, SOFA, Severe sepsis

## Abstract

**Background and the purpose of the study:**

sepsis is one of the most widespread and lethal disease in Intensive Care Units (ICU). Based on pathophisyology of sepsis, it seems that routine laboratory tests combined with analysis of pro-inflammatory cytokines plasma levels, help clinicians to have more information about disease progress and its correct management.

**Methods:**

This was a prospective observational study to determine the predictive role of Tumor Necrosis Factor alpha (TNF-α), Interleukin (IL)-1β and IL-6 as three main pro-inflammatory cytokines and Acute Physiology and Chronic Health Evaluation (APACHE II) and Sequential Organ Failure Assessment (SOFA) as two scoring systems in mortality of critically ill patients with severe sepsis. Fifty and five patients with criteria of severe sepsis were included in this study. An exclusion criterion was post Cardiopulmonary Resuscitation (CPR) status. Cytokines (TNF-α, IL-1β and IL-6) were assayed in the first, third and seventh days in blood of patients.

**Results and major conclusion:**

Among three measured cytokines, sequential levels of TNF-α and IL-6 showed significant differences between survivors and nonsurvivors. IL-6 had a good correlation with outcome and scoring systems during the period of this study. The areas under the receiver operating characteristic (AUROC) curve indicated that APACHE II (0.858, 0.848, 0.861) and IL-6 (0.797, 0.799, 0.899) had discriminative power in prediction of mortality during sequental measured days. Multiple logestic regression analysis identified that evaluation of APACHE II and TNF-α in the first day and APACHE II and IL-6 in the third and seventh days of severe septic patients are independent outcome predictors. Results of this study suggest that IL-6 and APACHE II are useful cytokine and scoring systems respectively in prediction of mortality and clinical evaluation of severe septic patients.

## INTRODUCTION

Sepsis is one of the most widespread and lethal disease in intensive care units which also charges the health system a lot (1). At present, judgment about outcome of septic patients and evaluation of treatment is based on clinical evaluation and using severity scores (2). It seems that routine laboratory and clinical examination do not accurately distinguish severity of illness and do not sensitively identify the risk of mortality in the septic patients. Recent advances on the immunologic processes and pathophysiology of sepsis help to use more sensitive and specific tools for determination of severity of sepsis and evaluation of disease management (3). Investigators have been trying to clarify the role of cytokines in various illnesses (4, 5). Extensive releases of various inflammatory mediators in response to pathogens cause catastrophic exacerbation of patients in severe sepsis and septic shock. Among cytokines, TNF-α, IL-1β and IL-6 play central roles for initiation of the innate immune response and propagation of acquired immune responses. Exaggerate activation of innate immunity with these cytokines can lead to vascular collapse, shock, and death (6). Early intervention to prevent clinical deterioration is the most important step in the successful management of patients with severe sepsis (7). Pursuit of management is a major obstacle in severe septic patients. The clinician should be able to realize the improvement of disease as soon as possible in order to refine treatment strategy and if it is possible the clinician should be equipped with diagnostic molecular tests which resound pathophysiological events (8). Based on previous surveys, the prognostic value of cytokines in septic patients remains controversial (9, 10). While a number of biomarkers have been, introduced but, it is not clear that measurement of which cytokine(s) are valuable and practical in clinical setting for prediction of outcome. The other important question is that which cytokine(s) is trustworthy for evaluation of severity of disease during management of illness to guide physicians to correct their treatments. There are several valuable studies which have focused to answer these two questions. In one of very recent published article (11), nine biomarkers were investigated. However like most of other articles published in this area, sequential changes of biomarkers were not considered. For answering to second question it was required to evaluate the level of sequential changes of biomarkers in plasma. Based on this hypothesis, prognostic value of the sequential plasma levels of the main pro-inflammatory cytokines (TNF-α, IL-1β and IL-6) and two scoring physiological systems (APACHE II and SOFA) in well-documented severe septic patients were investigated.

## MATERIAL AND METHODS

### 

#### Study design and setting

This was a prospective observational study which was conducted for 18 months in two surgico/medical intensive care units (ICU) of two general tutorial university hospitals.

#### Patient population

Fifty and one patients were included with severe sepsis criteria which was defined by the American College of Chest Physicians/Society of Critical Care Medicine Consensus Conference (12). Post CPR patients and patients with pre-existing conditions such as liver, lung diseases and malignancy were excluded. Study protocol was approved by the university ethics committee.

#### Method

Age, sex and cause of hospitalization were recorded on the admission of patients to the ICUs. Blood samples were collected in the first, third and seventh days after recruitment into the study to determine IL- 1β, IL-6 and TNF-α concentration. The APACHE II scores for definition of severity of illness and SOFA score as severity of organ failure were calculated in the first, third and seventh days of admission. Blood, urine and intratracheal cultures were obtained from all patients during hospitalization for identification of sepsis criteria to determine source and type of infection prior to initiation of antimicrobial therapy. For optimal sensitivity and specificity, blood cultures (aerobic and anaerobic bottles) were drawn from two or three different venipuncture sites. Standard treatment of sepsis included early goal- directed resuscitation, early broad - spectrum antibiotics, narrowing antibiotic therapy based on microbial therapy and clinical data, source control by surgical procedures, stress doses of steroids for septic shock, maintenance of blood glucose < 150 mg/dl, DVT/ stress ulcer prophylaxis, required homodynamic and ventilatory support were performed for patients enrolled in this study. The main outcome was 28-day mortality.

#### Cytokine measurement

Blood samples were collected between 10 and 12 A.M. using an arterial line for septic patients. Samples were centrifuged immediately in ICU at 800 g for 15 minutes and separated plasma was stored at -80°C. Plasma levels of TNF-α, IL-1β and IL-6 were measured by enzyme-linked immunosorbent assay (ELISA) with a commercial kit (Bender MedSystem Inc, Vienna, Austria) using the manufacture's protocol. All cytokine measurements were performed in a faculty laboratory blinded to the clinical data. The samples were assayed in duplicates with suitable controls provided by manufacturer for the construction of standard curves.

#### Statistics

Data are presented as mean ± standard error of mean. Univariate comparisons of baseline characteristics were performed by the independent Student's t-test in normally distributed data between two groups (survivors and non-survivors) and Mann-Whitney U test for non- normal data, respectively. Two-way analysis of variance was used to compare repeated measurements of cytokine levels between survivors and nonsurvivors with multiple comparisons (Scheffe's test) during time. A *P* value less than 0.05 was considered statistically significant. The correlations between outcome and the variables are reported as Spearman's rho correlation coefficient and among variables as Pearson's correlation coefficient. The multivariate analysis was performed by multiple logistic regression, using the “backward conditional” method for selection of the independent variables. The Odds Ratio (OR) of an independentvariable was calculated as OR = eb, where b was the regression coefficient. Receiver operating characteristic (ROC) curves were used to evaluate the diagnostic value for cytokines and scoring systems. Values for IL-6 and TNF-α in the first day were log transformed to obtain proportionally constant variation and distributed normally. Statistical analyses were performed using the SPSS 11.5 application (SPSS Inc., Chicago)

## RESULTS

### 

#### Population Characteristics

This prospective study enrolled 51 adult critically ill patients with criteria of severe sepsis. Of these patients, 25 died in the ICU and 26 patients survived. Detailed demographic, clinical, and microbiological information of survivors and non-survivors summarized are in table 1. The differences between age and sex were not significant in either group. Gram negative bacteria were the predominant microorganisms (76%) isolated in 42 out of 51 patients (82%). Blood culture was positive for near to half of patients (49%). For 22% of all patients, there was not any microbial etiology from any collection of specimens. All patients when enrolled in the study were mechanically ventilated. The number of missing data in the day of 3 and the day of 7 were 8 and 15, respectively. Mortality was the cause of missing data.

**Table 1:** Patients' characteristics

	Survivors	Non-survivors	*P* Value
No.	26	25	
Age (years)	46.7 ± 4.1	52.2 ± 3.7	* NS
Gender (Male/Female)	17/9	17/8	NS
Diagnose at entry:			
Multiple trauma	7	5	
Pneumonia	7	5	
Peritonitis	2	4	
Post surgical	4	2	
Cerebral disorders	4	5	
Others	2	4	
Microbiological etiology:			
Gram negative	19	13	
Gram positive	3	3	
Mixed gram negative/positive	1	3	
Unknown	3	6	
Source of infection detection:			
Blood	12	13	
Lung/urine/CSF	10	5	
Unknown	4	7	

* NS: Not significant

#### Scoring systems

Sequential changes in APACHE II and SOFA, two widespread scoring systems which were used in ICU are summarized in table 2. As expected, patients who died compared to survivors had higher APACHE II and SOFA scores. There were significant correlations between outcome with APACHE II (day 1: r = 0.621, p< 0.01, day 3: r = 0.592, p<0.01, day 7: r = 0.616, p<0.01) and SOFA (day 1: r = 0.361, p< 0.01, day 3: r = 0.451, p<0.01, day 7: r = 0.466, p<0.01) scores in all measured days. Among cytokines, only IL-6 had significant correlation with both APACHE-II (day 1: r = 0.588, p<0.01, day 7: r = 0.624, p<0.01) and SOFA (day 1: r = 0.533, day 3: r = 0.335, p<0.05, day 7: r = 0.700, p<0.01) scores.

**Table 2:** Scoring system changes in patients.

	Survivors	Non-survivors	*P* Value
Day 1			
APACHE II	17.7 ± 0.9	24.5 ± 1.0	0.000
SOFA	7.1 ± 0.5	9.5 ± 0.7	0.006
Day 3			
APACHE II	15.5 ± 1.1	23.1 ± 1.3	0.000
SOFA	5.8 ± 0.5	8.8 ± 0.8	0.002
Day 7			
APACHE II	14.2 ± 1.3	24.4 ± 1.7	0.000
SOFA	5.3 ± 0.5	8.9 ± 0.9	0.001

APACHE, Acute Physiology and Chronic Health Evaluation; SOFA, Sequential Organ Failure Assessment.

#### Cytokine concentrations

**Figure 1:**
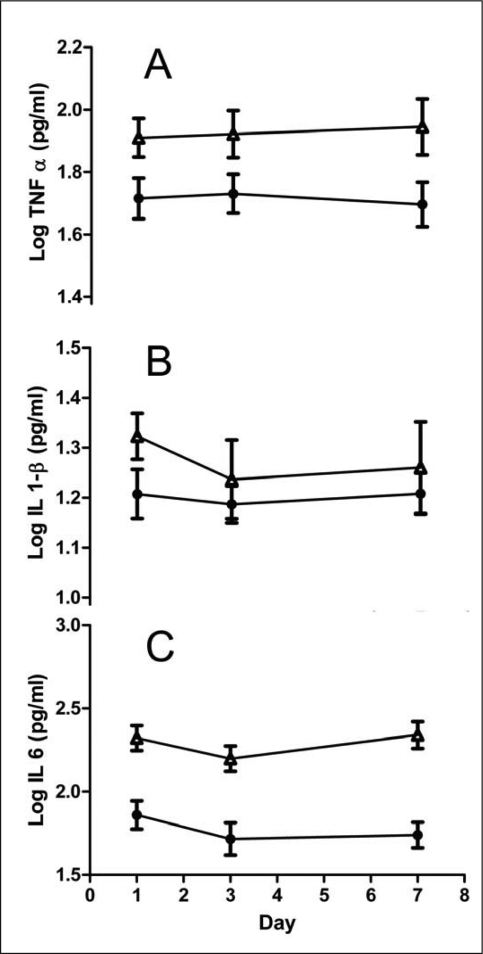
Changes in plasma concentration of TNF-α (A), IL-1β (B) and IL-6 (C) for survivors vs. nonsurvivors by day. Solid circles, survivors; open triangles, nonsurvivors. This figure shows that plasma level of log TNF-α and log IL-6 during days are significantly higher in nonsurvivors (p=0.043, p<0.01).

The cytokine plasma concentrations of survivors and nonsurvivors are shown in table 3. Cytokines levels in non-survivors were higher than survivors but in the first day, plasma level of TNF-α and IL-6 and in other days only IL-6 level showed significant differences between both groups. There were significant correlations between IL-6 and TNF-α on the day of 3 (r = 0.389, p<0.01) and 7 (r = 0.0349, p<0.05) and between IL-6 and IL-1β on the day of 3 (r = 0.466, p<0.01). Outcome had correlation with IL-6 (day 1: r = 0.514, p<0.01, day 3: r = 0.506, p<0.01, day 7: r = 0.652, p<0.01), and TNF-α (day 1: r = 0.278, p<0.05, day 3: r = 0.312, p<0.05, day 7: r = 0.329, p<0.05) in all measured days but did not show any significant correlation with IL-1β. There was no significant difference in sequential changes in plasma level of cytokines for IL-6 (p <0.01) and TNF-α (p=0.043) (Figure 1).

**Table 3:** Cytokines concentration

	Survivors	Non-survivors	*P* Value
Day 1			
TNF-α	70.1 ± 12.2	102.9 ± 15.4	0.049*
IL-6	102.9 ± 14.4	283.8 ± 40.4	0.000*
IL1-β	19.0 ± 2.3	23.9 ± 2.4	0.138
Day 3			
TNF-α	69.1 ± 9.6	102.4 ± 14.3	0.051
IL-6	84.5 ± 15.2	202.9 ± 39.9	0.003
IL1-β	16.8 ± 1.4	22.1 ± 3.8	0.210
Day 7			
TNF-α	67.3 ± 11.0	105.0 ± 15.2	0.054
IL-6	79.1 ± 14.9	260.3 ± 43.1	0.000
IL1-β	18.1 ± 2.1	23.0 ± 4.7	0.273

* a Mann-Whitney rank sum test, Values are in pg/ml , IL, interleukin; TNF, tumor necrosis factor.

#### Predictive values of cytokines and scoring systems for severe sepsis mortality

Areas under ROC curves were calculated to analyze the discriminative power of cytokine levels and scoring systems for the prediction of severe sepsis mortality. The AUROC closer to 1 indicates greater discriminatory power, whereas an AUROC of less than 0.5 implies no diagnostic potential. Among cytokines, the highest AUC belonged to IL-6 which was the best predictor of mortality in all measured days. TNF-α had significant relation in the prediction of mortality but it was weaker than IL-6. IL-1β and did not show relation with prediction of outcome in any time intervals (Table 4). Both of scoring systems showed suitable trends in the prediction of mortality but as shown in table 4, APACHE II was the best one in the prediction of outcome in the course of time. Variables in multiple stepwise logistic regression analysis were included with backward elimination method to find out the fittest model and independent variables for prediction of mortality. The odds ratio is an estimate of the increase (or decrease) in the odds for outcome if the independent variable value is increased by 1. In the first day only TNF-α and APACHE II were introduced as a best predictors in final model (Table 5) but in the days of 3 and 7 IL-6 and APACHE II independently discriminated vital status. In a stepwise analysis, IL-6 was removed from model of the first day in the latest step.

**Table 4:** ROC curve analysis

Variable	AUROC (95%CI)	*P* Value
Day 1		
TNF-α	0.661 (.509–.812)	0.049
IL-6	0.797 (.675–.918)	0.000
IL-β	0.628 (.473–.782)	0.118
APACHE II	0.858 (.752–.964)	0.000
SOFA	0.707 (.563–.851)	0.074
Day 3		
TNF-α	0.684 (.521-.848)	0.043
IL-6	0.799 (.669–.928)	0.001
IL-β	0.559 (.364–.753)	0.518
APACHE II	0.848 (.734–.936)	0.000
SOFA	0.765 (.622–.908)	0.004
Day 7		
TNF-α	0.701 (.514–.889)	0.052
IL-6	0.899 (.801–.998)	0.000
IL-β	0.552 (.312–.792)	0.615
APACHE II	0.861 (.740–.982)	0.000
SOFA	0.792 (.621–.962)	0.005

AUROC, area under the receiver operating characteristic curve; CI, confidence interval; IL, interleukin; TNF, tumor necrosis factor; APACHE, Acute Physiology and Chronic Health Evaluation; SOFA, Sequential Organ Failure Assessment.

**Table 5:** Final models built using stepwise logistic regression to predict mortality during cytokines and scoring systems measured days.

	Coefficient	Odds ratio (95% CI)	*P* Value
Day 1			
APACHE II	0.375	1.455 (1.177–1.799)	0.001
TNF-α	0.012	1.012 (.999–1.025)	0.061
Constant	−8.883	0.000	0.000
Day 3			
APACHE II	0.244	1.267 (1.080-1.508)	0.004
IL-6	0.009	1.009 (1.001–1.018)	0.033
Constant	−6.474	0.002	0.001
Day 7			
APACHE II	0.187	1.206 (.988–1.471)	0.065
IL-6	0.012	1.012 (1.001–1.024)	0.036
Constant	−6.041	0.002	0.008

APACHE, Acute Physiology and Chronic Health Evaluation; SOFA, Sequential Organ Failure Assessment; IL, interleukin; TNF, tumor necrosis factor; CI, confidence interval.

## DISSCUTION

It has been shown that TNF-α, IL-1β and IL-6 have main role in inflammation caused by infection (13). Adapted to this pattern, it was also found that proinflammatory cytokines were elevated in the plasma of septic patients. Sequential plasma levels of IL-6 and TNF-α elevated significantly in non- survivors. Many of the fundamental responses to acute inflammation are mediated, in part, by TNF-α production which is a mediator of both innate and specific immunity and an important trigger for acute inflammatory response. Results of this study showed that continuous elevated level of TNF-α makes the inflammation status rambunctious. Correlation among cytokines in the third and seventh days showed that they augment each other's magnitude. In addition, the strong correlation between physiological scoring systems and outcome with cytokines in the course of the study proved that critical illness was associated with elevated level of cytokines.

IL-6 compared to TNF-α, levels was higher than baseline and more significant than TNF-α in non- survivors during measured days and due to its stable kinetic in plasma, it could be justified that IL-6 is a valuable cytokine for prediction of outcome in sepsis setting which is in agreement with previous reports (10, 14, 15). On the other hand, due to strong correlation of IL-6 with scoring systems in all of measured days, level of IL-6 is also trustworthy for evaluation of severity of sepsis. But in this regard, a few previous reported studies have not confirmed the mortality predictive ability of cytokines in septic patients (9, 16, 17).

In agreement with other investigations, it was found that non-survivors did not have significant higher level of IL-1β compared to survivors (14, 17). However result of another study has shown that IL-1β was higher in dead patients and it can be used as an early marker for outcome prediction (10).

These finding were also evaluated and approved with ROC performance. Because of higher AUC of IL-6 compared to other two cytokines in all measured days, IL-6 may be suggested as a suitable cytokine with a high discriminative power in the prediction of mortality during time. It has been reported that the production of cytokines in response to stress varies among ethnic population, probably because of cytokines gene polymorphism (18). Therefore, in AUROC performance, we didn't report the best cutoff point with ideal sensitivity and specificity for plasma level of cytokines. Therefore this problem seems to be one of the most challenging topics in the future of cytokines measurement as an applied clinical test. It is believed that after general agreement on a selected cytokine(s) for measurement in clinic, the cutoff point should be determined for every ethnic population independently with acceptable numbers of patients or a multi national study should be set for determination of an acceptable cutoff point.

Since APACHE II reflects magnitude of the physiological response and SOFA shows organ dysfunction, using cytokines for evaluation of inflammatory response may improve accurate prediction of outcome. Therefore in the present study, a multiple logistic regression analysis was performed to examine whether any of cytokines along with scoring systems have contribution to the final model for mortality prediction. IL-6 showed stronger correlation with mortality than TNF-α in all measured days which was also confirmed by AUROC curves. However results of multivariate analysis showed that TNF-α and APACHE II score in the first day and IL-6 in accompany with APACHE II score in the third and seventh days were independently correlated with outcome (Table 4). Although IL-6 was removed from the model of the first day in the latest step of stepwise analysis (Table 5) it seems that IL-6 may have a potent independent relation in the prediction of outcome even for the first day. Some studies which determined independent predictors of outcome by performing multiple logistic regressions and included TNF-α, IL-1β and IL-6 as measured cytokines, have introduced IL-6 as independent factors (15, 19). Procalcitonin (PCT), a protein with identical sequence as the precursor protein of the human calcitonin hormone, is a new diagnostic parameter of bacterial infection and systemic inflammation. High plasma concentrations of PCT have been found in patients with severe bacterial and fungal infections, in sepsis and in patients suffering from multi organ dysfunction syndrome (MODS). Several clinical studies aimed to establish the usefulness of PCT to confirm the diagnosis and to predict outcome of septic patients (20).

Thus, for future studies, measurements of plasma PCT concentrations may be a helpful approach to improve the outcome prediction in patients with severe sepsis particularly when combined with the APACHE II scoring system and IL-6 measurement. Other than sepsis, a number of etiologies cause inflammation in critically ill patients. While specificity in correct diagnosis is important, sensitivity to monitor treatment sufficiency is also critical. It seems that finding of biomarker with high specificity and sensitivity will have a strategic role in management of septic patient.

## CONCLUSION

Measurement of TNF-α and IL-6 at the early phase of sepsis may be helpful to evaluate severity of disease and to estimate outcome. Plasma level of IL-6 can be measured to monitor treatment sufficiency. It is believed that combination of these two cytokines with APACHE II score is valuable indicator in management of septic patients.
